# Investigating the role of aspirin on the mortality risk of sepsis-associated encephalopathy: a retrospective study

**DOI:** 10.3389/fneur.2025.1521043

**Published:** 2025-03-26

**Authors:** Fengzhen Huang, Jiping Yi, Qiuli Li, Tieqiao Zhou, Dongyou Yue, Xiaoxiang Gong, Heng Meng

**Affiliations:** ^1^Department of Neurology and Stroke Center, The First Affiliated Hospital of Jinan University, Guangzhou, Guangdong, China; ^2^Department of Neurology, The First People’s Hospital of Chenzhou Affiliated to the University of South China, Chenzhou, Hunan, China; ^3^Department of Neurology, The First Affiliated Hospital of Xiangnan University, Chenzhou, Hunan, China; ^4^Department of Laboratory Medicine, The First People’s Hospital of Chenzhou Affiliated to the University of South China, Chenzhou, Hunan, China; ^5^Department of Emergency, The First People’s Hospital of Chenzhou Affiliated to the University of South China, Chenzhou, Hunan, China; ^6^Department of Pediatrics, The Second Xiangya Hospital, Central South University, Changsha, Hunan, China

**Keywords:** sepsis, sepsis-associated encephalopathy (SAE), aspirin, MIMIC IV database, mortality

## Abstract

**Background:**

Sepsis-associated encephalopathy (SAE) is one of the most common complications of sepsis. Aspirin can serve as a promising therapeutic candidate and help improve patient outcomes in sepsis and its complications. However, the efficacy and safety of aspirin on SAE remains largely unexplored.

**Methods:**

Patients for this retrospective study were collected from MIMIC-IV (version 3.0). Propensity score matching (PSM) was used to balance the baseline characteristics between the no aspirin group and aspirin group. The association between aspirin therapy and mortality risk of in-hospital, 30-day, 60-day, 90-day, and 180-day was analyzed by Cox proportional hazards model and Kaplan–Meier method. *E*-value analysis was used to evaluate the potential influence of unmeasured or unknown confounding factors. Subgroup analysis was applied to explore potential differences in the effects of aspirin therapy on clinical outcomes across these various groups.

**Results:**

Our study recruited 4,707 SAE patients in total, and 2,518 patients were enrolled after PSM. The mortality rate for in-hospital, 30-day, 60-day, 90-day, and 180-day in the aspirin group was consistently significant lower than that in the no aspirin group. Kaplan–Meier curves revealed that the SAE patients received aspirin therapy exhibited a notably higher survival rate compared to those who did not. The risk of gastrointestinal hemorrhage had no significant difference between the two groups. Additionally, the mortality rate of SAE patients in aspirin pre-ICU group, aspirin in-ICU group, and aspirin pre-ICU and in-ICU group decreased significantly compared to the no aspirin group. The high-dose aspirin group experienced a significantly higher mortality rate compared to those in the low-dose group.

**Conclusion:**

Aspirin could reduce the mortality risk of SAE patients for in-hospital, 30-day, 60-day, 90-day, and 180-day, without increasing the risk of gastrointestinal hemorrhage. The benefits observed persisted regardless of aspirin exposure timing. Patients received high-dose aspirin exhibited a higher mortality risk compared to those in the low-dose group.

## Introduction

Sepsis is a life-threatening syndrome characterized by a dysregulated host response to infection. The overwhelming inflammatory reaction leads to multiple organ dysfunction and failure, and the mortality rate escalates rapidly without prompt and appropriate treatment (1, 2). Sepsis-associated encephalopathy (SAE) is one of the most common complications of sepsis, affecting approximately 70% septic patients. It presents as a widespread brain malfunction induced by the systemic response to infection, without direct clinical or laboratory evidence indicating brain infection or other types of encephalopathy, such as hepatic encephalopathy or renal encephalopathy. The factors contributing to brain dysfunction in sepsis include neurodegeneration, neuroinflammation, changes in neurotransmission, disruption of the blood–brain barrier, and impaired cerebral microcirculation (3, 4). SAE patients exhibit a wide range of clinical presentations, varying from subtle behavioral alterations to significant cognitive decline, and subsequently to disruptions of consciousness and coma (5, 6). The mortality risk increases in patients experiencing SAE, and those who survive may endure persistent neurological and psychological impairments (7, 8). However, there exists a shortage of effective therapeutic options for clinical management on this serious condition.

Aspirin was a traditional non-selective cyclooxygenase (COX) inhibitor, and it has been extensively used in the treatment and prevention strategy of cardiovascular and cerebrovascular diseases, due to its capacity of inhibiting platelet aggregation (9). Recent researches have shed light on an expanded range of potential benefits for aspirin, especially in sepsis. Aspirin can serve as a promising therapeutic candidate to help improve patient outcomes by modulating the excessive inflammation in sepsis (10, 11). The anti-inflammatory effect of aspirin may be related to the irreversible acetylation of cyclooxygenase enzymes (12). Research found that the 90-day mortality rate in the aspirin group was significantly lower compared to the no-aspirin group, indicating that administering aspirin within 24 h of ICU admission was associated with reduced 90-day mortality in septic patients (13). It has also been demonstrated to promote recovery in patients suffering from sepsis-related complications, including acute kidney injury, acute respiratory failure, acute respiratory distress syndrome (ARDS), and sepsis-induced myocardial injury. All these conditions are common complications of sepsis with high morbidity and mortality (14–19). Despite these promising findings, the potential benefits of aspirin on SAE remains largely unexplored. It is essential for future research that focus on exploring aspirin’s potential neuroprotective effects on SAE.

Therefore, we aim to conduct a real-world study with MIMIC-IV database to evaluate the possible efficacy and safety of aspirin exposure on SAE patients. We consider that aspirin could reduce the mortality risk in SAE patients for in-hospital, 30-day, 60-day, 90-day, and 180-day.

## Methods

### Data source

We used data from MIMIC-IV database [version 3.0 (20, 21)] for this retrospective cohort study. MIMIC-IV comprised detailed clinical information of patients spanning from 2008 to 2022 by two in-hospital database systems: a customized hospital-wide electronic health record (EHR) and an intensive care unit (ICU). The author Fengzhen Huang successfully passed the Collaborative Institutional Training Initiative (CITI) exam and obtained the permission to access the database (Record ID: 63858817 and 63858818). The database utilization was approved by the Institutional Review Boards of the Massachusetts Institute of Technology and Beth Israel Deaconess Medical Center. The data has been de-identified and informed consent was thus not necessary.

### Study population

MIMIC-IV database contained a total of 364,627 patients, 546,028 admissions, and 94,458 icustays. The sepsis was defined as a suspected infection combined with an acute increase in Sequential Organ Failure Assessment (SOFA) score ≥ 2 (22). Patients who met both of the following criteria could be defined as SAE: (1) patients with sepsis; (2) patients having a score on the Glasgow Coma Scale (GCS) < 15 or having delirium (23). GCS has been used to identify SAE and distinguish it from sepsis. It was assessed prior to sedation or surgery for the relevant patients. Delirium was diagnosed according to the International Classification of Diseases codes (ICD) version 9 or 10. Among these SAE patients, the inclusion criteria included: (1) First ICU admission; (2) ICU stay duration >24 h; (3) Age ≥ 18 years. The exclusion criteria included: (1) Patients with primary brain injury, including traumatic brain injury, epilepsy, brain tumor, meningitis or encephalitis, and cerebrovascular disease; (2) Patients with mental disorders; (3) Patients with neurodegenerative diseases; (4) Patients with alcohol or drug abuse; (5) Patients with metabolic encephalopathy, hepatic encephalopathy, hypertensive encephalopathy, hypoglycemic coma, and other liver or kidney diseases affecting consciousness; and (6) Patients with severe electrolyte imbalances or glycemic disturbances, including hyponatremia (<120 mmol/L), hypernatremia (>150 mmol/L), hypoglycemia (<54 mg/dL), hyperglycemia (>180 mg/dL); (7) Patients with insufficient data (missing data >20%).

### Data collection

The patient information were extracted using Structured Query Language (SQL). The following variables were collected: (1) demographic information: age, gender, race, weight; (2) laboratory indexes within the first day of hospitalization: wbc, hemoglobin, platelet, glucose, sodium, potassium, calcium, chloride, creatinine, aniongap, bun, partial thromboplastin time (PTT), prothrombin time (PT), po2, pco2, base excess; (3) mean values of vital signs within the first day of ICU: Saturation of Peripheral Oxygen (Spo2) mean, systolic blood pressure (SBP) mean, diastolic blood pressure (DBP) mean, heart rate mean, respiratory rate (Resp rate) mean, temperature mean; (4) disease severity scores at the time of ICU admission: Sepsis-related Organ Failure Assessment (SOFA), Glasgow Coma Scale (GCS), Acute Physiology Score III (APSIII), Objective Structured Assessment of Intensive Care Skills (OASIS), Charlson Comorbidity Index (CCI); (5) comorbidities diagnosed via ICD codes 9 or 10: hypertension, diabetes, heart failure, cardiovascular disease, chronic obstructive pulmonary disease (COPD); and (6) therapeutic interventions during hospitalization: continuous renal replacement therapy (CRRT), vasoactive agent, ventilation.

### Outcomes

Participants were divided into two groups: no aspirin group and aspirin group. Aspirin exposure was defined as receiving at least one dose of any form of aspirin during hospitalization. In addition, we divided SAE patients into the following four groups to investigate the association between the clinical outcome and different aspirin exposure timing: aspirin pre-ICU group, aspirin in-ICU group, aspirin pre-ICU and in-ICU group, and no aspirin group. We also compared the outcome between the low-dose aspirin group (<300 mg/d) and high-dose aspirin group (> = 300 mg/d).

The primary outcome was 30-day mortality. The secondary outcomes included in-hospital mortality, 60-day mortality, 90-day mortality, 180-day mortality, length of hospital stay and gastrointestinal hemorrhage. The complication of gastrointestinal hemorrhage was diagnosed according to ICD-9 or 10.

### Statistical analysis

All baseline variables had fewer than 20% missing data, which was randomly imputed using the “mice” package in R software. Since the skewed distribution of continuous variables, the comparisons were made by Mann–Whitney U test and presented in the form of the median and inter-quartile range (IQR). Categorical variables were compared by the chi-square test and expressed as numbers and percentages.

Propensity score matching (PSM) was used to balance the baseline characteristics between the no aspirin group and aspirin group (24). We performed PSM by nearest neighbor matching, with a caliper width of 0.05 and patients matched at a ratio of 1:1. The balance of the variables was assessed by the standardized mean difference (SMD), and it was considered balanced if the SMD < 0.1 between the two groups.

The Cox proportional hazards model, with hazard ratio (HR) and 95% confidence interval (CI), was applied to assess the association between aspirin therapy and mortality risk of in-hospital, 30-day, 60-day, 90-day, and 180-day. The Kaplan–Meier survival curves were generated and analyzed by the log-rank test. The multivariate models were adjusted for various variables. *E*-value analysis was used to evaluate the potential influence of unmeasured or unknown confounding factors. This analysis aimed to evaluate the extent of confounding effects needed to eliminate the observed association between aspirin administration and mortality risk (25). Subgroup analysis was applied to explore potential differences in the effects of aspirin therapy on clinical outcomes across these various groups. In addition, we conducted a assessment between the aspirin exposure timing and mortality rate. We also did a evaluation between the dosage of aspirin and mortality rate. All statistical analysis were conducted using R software (v4.4.0), with statistical significance determined by a two-tailed *p*-value <0.05.

## Results

### Baseline characteristics

**Figure 1:**
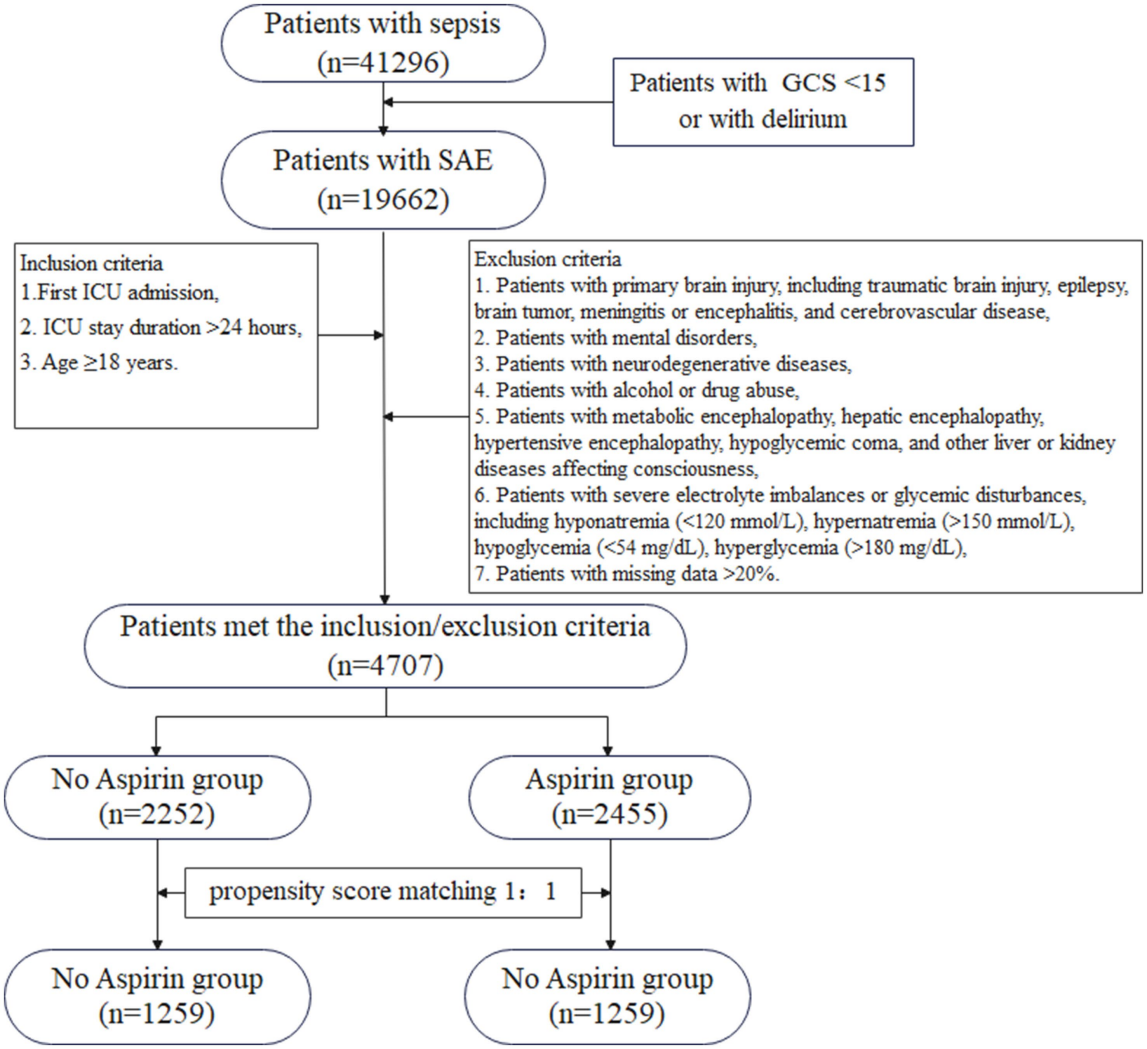
The flowchart for the selection of SAE patients. GCS, Glasgow Coma Scale; SAE, sepsis-associated encephalopathy; ICU, intensive care unit.

**Figure 2:**
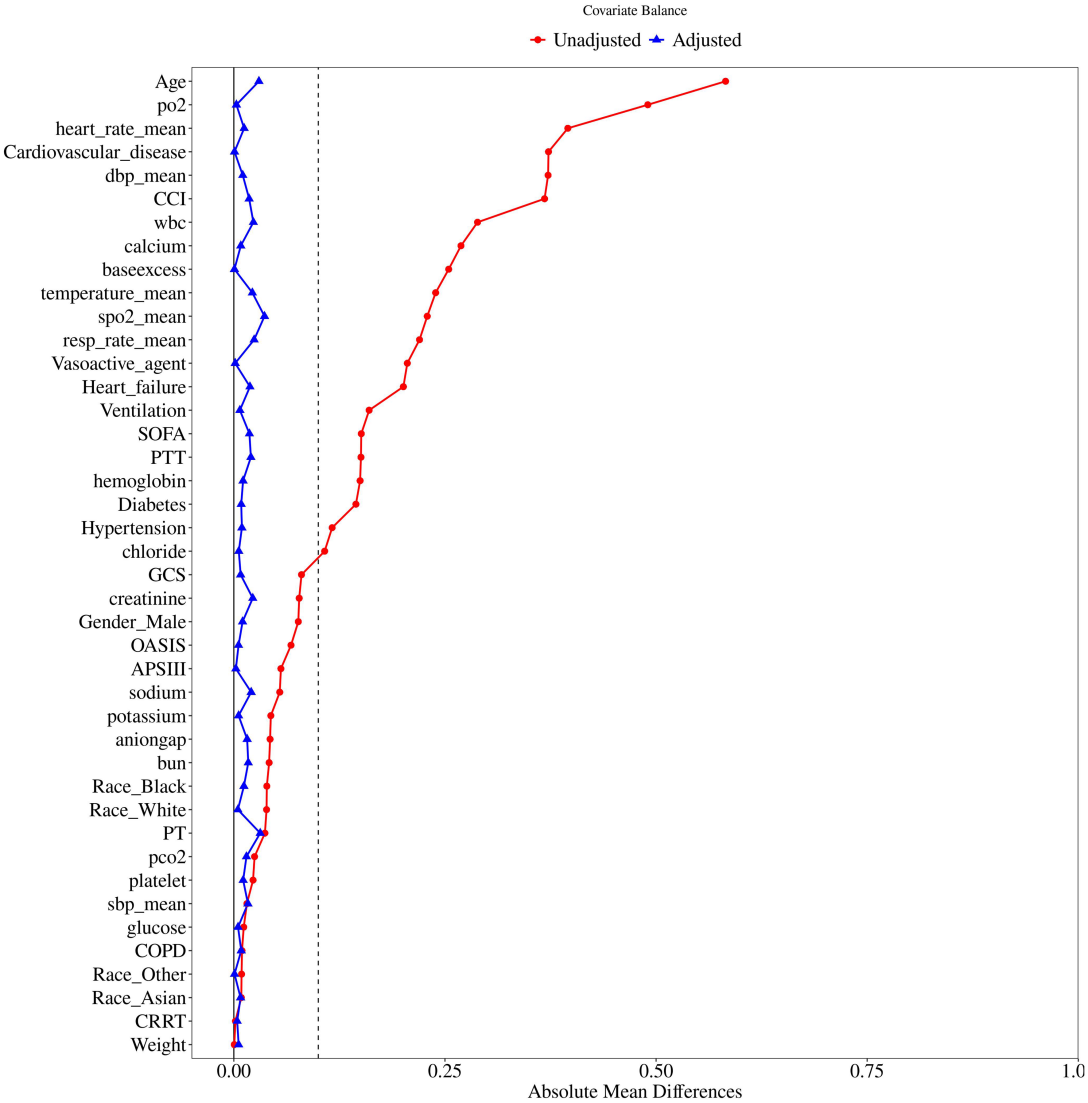
The difference of baseline characteristics between the two groups.

Figure 1 illustrated the flowchart for selecting SAE patients. In total, 41,296 patients were diagnosed with sepsis, and a subset of 19,662 patients met the SAE criteria in the MIMIC-IV database. Subsequently, 4,707 eligible patients were recruited after the inclusion and exclusion criteria. Participants were divided into two groups: no aspirin group, which included individuals who did not receive aspirin treatment (*n* = 2,252), and the aspirin group, which included those who received aspirin during hospitalization (*n* = 2,455). As a retrospective study, there were notable differences in most baseline characteristics between the two groups. Compared to the no aspirin group, patients in the aspirin group were older, heavier, and had a higher proportion of males. In the aspirin group, the hemoglobin, BUN, and PO2 levels were higher, while PT was shorter, compared to the no aspirin group. The WBC, potassium, calcium, chloride, creatinine, and PTT in both groups were within the normal range. The aspirin group also exhibited a higher prevalence of chronic comorbidities (hypertension, diabetes, heart failure, cardiovascular disease), as well as higher OASIS and CCI scores. Propensity score matching (PSM) was employed to alleviate the interference from confounders. A total of 2,518 patients were screened, with 1,259 in each group after PSM. The Standardized Mean Differences (SMD) for all variables were < 0.1, and all *p*-values were > 0.05, indicating that the baseline characteristics were well balanced between the two groups (Table 1). Figure 2 depicted the difference of baseline characteristics between the two groups before and after PSM.

**Table 1:** Baseline characteristics of SAE patients before and after PSM.

Variable	Before PSM					After PSM				
	Total (*n* = 4,707)	No aspirin (*n* = 2,252)	Aspirin (*n* = 2,455)	*p*	SMD	Total (*n* = 2,518)	No aspirin (*n* = 1,259)	Aspirin (*n* = 1,259)	*p*	SMD
Demographics										
Age, M (Q₁, Q₃)	71.76 (60.64, 81.38)	67.56 (54.20, 80.28)	74.63 (65.26, 82.19)	**<0.001**	0.582	73.69 (62.65, 82.52)	73.86 (62.39, 83.34)	73.44 (62.81, 82.02)	0.731	0.027
Weight, M (Q₁, Q₃)	77.00 (64.35, 91.10)	76.00 (63.00, 91.03)	77.30 (65.70, 91.35)	**0.012**	0.000	76.80 (63.50, 91.00)	76.80 (62.90, 91.35)	76.90 (64.15, 90.80)	0.623	−0.005
Gender, n (%)				**<0.001**					0.601	
Female	2,001 (42.51)	1,047 (46.49)	954 (38.86)		−0.157	1,101 (43.73)	544 (43.21)	557 (44.24)		0.021
Male	2,706 (57.49)	1,205 (53.51)	1,501 (61.14)		0.157	1,417 (56.27)	715 (56.79)	702 (55.76)		−0.021
Race, n (%)				**<0.001**					0.565	
Asian	156 (3.31)	85 (3.77)	71 (2.89)		−0.053	90 (3.57)	50 (3.97)	40 (3.18)		−0.045
Black	458 (9.73)	265 (11.77)	193 (7.86)		−0.145	249 (9.89)	117 (9.29)	132 (10.48)		0.039
Other	800 (17)	372 (16.52)	428 (17.43)		0.024	407 (16.16)	203 (16.12)	204 (16.20)		0.002
White	3,293 (69.96)	1,530 (67.94)	1,763 (71.81)		0.086	1,772 (70.37)	889 (70.61)	883 (70.14)		−0.010
Laboratory index										
Wbc, M (Q₁, Q₃)	9.80 (7.10, 14.20)	10.60 (7.30, 15.40)	9.30 (6.95, 13.10)	**<0.001**	−0.289	10.10 (7.20, 14.40)	10.20 (7.30, 14.65)	10.00 (7.15, 14.20)	0.360	0.019
Hemoglobin, M (Q₁, Q₃)	10.60 (9.00, 12.30)	10.50 (8.80, 12.10)	10.70 (9.20, 12.40)	**<0.001**	0.150	10.40 (8.90, 12.00)	10.40 (8.85, 12.00)	10.40 (8.90, 12.00)	0.790	0.011
Platelet, M (Q₁, Q₃)	203.00 (148.00, 274.00)	207.00 (144.00, 286.00)	201.00 (151.00, 263.50)	0.412	−0.023	204.00 (147.00, 275.00)	206.00 (145.00, 280.50)	201.00 (148.50, 270.50)	0.611	−0.010
Glucose, M (Q₁, Q₃)	116.00 (98.00, 138.00)	117.00 (98.00, 138.00)	116.00 (98.00, 139.00)	0.962	0.012	117.00 (98.00, 139.00)	118.00 (98.00, 139.00)	117.00 (97.00, 139.00)	0.965	0.005
Sodium, M (Q₁, Q₃)	139.00 (136.00, 141.00)	139.00 (136.00, 141.00)	139.00 (136.00, 141.00)	0.313	0.054	139.00 (136.00, 141.00)	139.00 (136.00, 141.00)	139.00 (136.00, 141.00)	0.804	0.020
Potassium, M (Q₁, Q₃)	4.10 (3.70, 4.50)	4.10 (3.70, 4.50)	4.10 (3.80, 4.50)	**0.002**	0.044	4.10 (3.70, 4.50)	4.10 (3.70, 4.50)	4.10 (3.70, 4.50)	0.430	0.005
Calcium, M (Q₁, Q₃)	8.50 (8.00, 9.00)	8.40 (7.90, 8.90)	8.60 (8.10, 9.10)	**<0.001**	0.269	8.50 (8.00, 9.00)	8.40 (7.90, 8.90)	8.50 (8.00, 9.00)	0.291	0.008
Chloride, M (Q₁, Q₃)	103.00 (100.00, 106.00)	103.00 (100.00, 107.00)	103.00 (99.00, 106.00)	**<0.001**	−0.108	103.00 (100.00, 107.00)	103.00 (100.00, 107.00)	103.00 (100.00, 107.00)	0.828	0.006
Creatinine, M (Q₁, Q₃)	1.00 (0.80, 1.50)	1.00 (0.70, 1.50)	1.10 (0.80, 1.60)	**<0.001**	0.077	1.10 (0.80, 1.70)	1.00 (0.70, 1.60)	1.10 (0.80, 1.70)	0.093	0.021
Aniongap, M (Q₁, Q₃)	14.00 (12.00, 16.00)	14.00 (12.00, 16.00)	14.00 (12.00, 16.00)	0.360	−0.043	14.00 (12.00, 16.00)	14.00 (12.00, 16.00)	14.00 (12.00, 16.00)	0.755	0.015
Bun, M (Q₁, Q₃)	21.00 (14.00, 35.00)	20.00 (13.00, 34.00)	22.00 (16.00, 36.00)	**<0.001**	0.042	22.00 (15.00, 37.00)	22.00 (15.00, 37.00)	22.00 (15.00, 38.00)	0.275	0.016
PTT, M (Q₁, Q₃)	31.10 (27.60, 36.90)	30.60 (27.20, 35.80)	31.50 (28.00, 37.80)	**<0.001**	0.151	30.90 (27.50, 36.50)	31.00 (27.45, 37.00)	30.90 (27.70, 35.90)	0.768	−0.024
PT, M (Q₁, Q₃)	13.90 (12.40, 16.50)	14.10 (12.60, 16.70)	13.70 (12.30, 16.35)	**<0.001**	−0.037	14.00 (12.60, 16.90)	14.10 (12.60, 17.15)	13.90 (12.50, 16.80)	0.118	−0.031
Po2, M (Q₁, Q₃)	106.00 (63.00, 234.50)	91.00 (54.75, 163.00)	134.00 (72.00, 341.00)	**<0.001**	0.490	97.00 (61.00, 188.00)	101.00 (62.00, 190.50)	94.00 (61.00, 182.50)	0.311	0.004
Pco2, M (Q₁, Q₃)	41.00 (36.00, 47.00)	41.00 (35.00, 48.00)	41.00 (36.00, 47.00)	0.257	−0.025	41.00 (36.00, 48.00)	41.00 (36.00, 48.00)	41.00 (36.00, 47.00)	0.805	−0.013
Base excess, M (Q₁, Q₃)	0.00 (−3.00, 2.00)	0.00 (−4.00, 2.00)	0.00 (−2.00, 2.00)	**<0.001**	0.254	0.00 (−3.00, 2.00)	0.00 (−3.00, 2.00)	0.00 (−3.00, 2.00)	0.806	0.001
Vital signs										
Spo2 Mean, M (Q₁, Q₃)	97.08 (95.70, 98.33)	96.85 (95.44, 98.19)	97.24 (95.96, 98.46)	**<0.001**	0.229	96.94 (95.53, 98.26)	96.92 (95.53, 98.30)	96.96 (95.53, 98.25)	0.769	0.035
SBP Mean, M (Q₁, Q₃)	113.13 (104.64, 124.01)	113.24 (103.70, 125.29)	113.00 (105.43, 122.71)	0.834	−0.015	113.77 (104.33, 126.36)	113.92 (104.04, 126.23)	113.65 (104.67, 126.55)	0.788	0.015
DBP Mean, M (Q₁, Q₃)	59.76 (53.88, 66.47)	61.58 (55.54, 68.88)	58.07 (52.76, 64.24)	**<0.001**	−0.372	60.23 (54.07, 66.76)	60.42 (53.99, 67.06)	60.08 (54.19, 66.61)	0.760	−0.010
Heart Rate Mean, M (Q₁, Q₃)	85.28 (75.36, 96.79)	88.88 (77.50, 101.54)	82.50 (74.32, 92.22)	**<0.001**	−0.396	85.18 (74.70, 95.99)	85.28 (74.47, 96.42)	85.04 (74.94, 95.89)	0.940	−0.011
Resp Rate Mean, M (Q₁, Q₃)	19.04 (16.67, 22.04)	19.56 (16.76, 22.76)	18.72 (16.60, 21.40)	**<0.001**	−0.220	19.31 (16.73, 22.30)	19.32 (16.60, 22.27)	19.31 (16.92, 22.31)	0.481	0.022
Temperature Mean, M (Q₁, Q₃)	36.81 (36.56, 37.12)	36.86 (36.61, 37.22)	36.76 (36.52, 37.05)	**<0.001**	−0.239	36.80 (36.56, 37.12)	36.80 (36.57, 37.12)	36.80 (36.55, 37.11)	0.318	−0.021
Severity score										
SOFA, M (Q₁, Q₃)	3.00 (2.00, 4.00)	3.00 (2.00, 4.00)	3.00 (2.00, 4.00)	**<0.001**	0.151	3.00 (2.00, 4.00)	3.00 (2.00, 4.00)	3.00 (2.00, 4.00)	0.924	0.019
GCS, M (Q₁, Q₃)	14.00 (10.00, 14.00)	14.00 (10.00, 14.00)	14.00 (10.00, 14.00)	0.523	−0.080	14.00 (10.00, 14.00)	13.00 (10.00, 14.00)	14.00 (10.00, 14.00)	0.069	0.009
APSIII, M (Q₁, Q₃)	49.00 (36.00, 66.00)	50.00 (37.00, 65.25)	49.00 (36.00, 67.00)	0.293	−0.056	50.00 (38.00, 66.00)	50.00 (37.00, 66.00)	50.00 (39.00, 66.50)	0.402	0.002
OASIS, M (Q₁, Q₃)	35.00 (29.00, 41.00)	35.00 (29.00, 41.00)	36.00 (30.00, 41.00)	**0.001**	0.068	35.00 (29.00, 42.00)	35.00 (29.00, 42.00)	36.00 (29.00, 41.00)	0.720	−0.005
CCI, M (Q₁, Q₃)	5.00 (3.00, 7.00)	5.00 (2.00, 7.00)	6.00 (4.00, 8.00)	**<0.001**	0.368	6.00 (4.00, 8.00)	5.00 (4.00, 8.00)	6.00 (4.00, 7.00)	0.554	0.018
Comorbidities										
Hypertension, n (%)				**<0.001**					0.627	
No	2,793 (59.34)	1,473 (65.41)	1,320 (53.77)		−0.233	1,486 (59.02)	749 (59.49)	737 (58.54)		−0.019
Yes	1,914 (40.66)	779 (34.59)	1,135 (46.23)		0.233	1,032 (40.98)	510 (40.51)	522 (41.46)		0.019
Diabetes, n (%)				**<0.001**					0.619	
No	3,445 (73.19)	1,818 (80.73)	1,627 (66.27)		−0.306	1,853 (73.59)	932 (74.03)	921 (73.15)		−0.020
Yes	1,262 (26.81)	434 (19.27)	828 (33.73)		0.306	665 (26.41)	327 (25.97)	338 (26.85)		0.020
Heart failure, n (%)				**<0.001**					0.319	
No	3,098 (65.82)	1,718 (76.29)	1,380 (56.21)		−0.405	1,614 (64.1)	819 (65.05)	795 (63.15)		−0.040
Yes	1,609 (34.18)	534 (23.71)	1,075 (43.79)		0.405	904 (35.9)	440 (34.95)	464 (36.85)		0.040
Cardiovascular disease, n (%)				**<0.001**					0.958	
No	3,322 (70.58)	2,027 (90.01)	1,295 (52.75)		−0.746	2,075 (82.41)	1,038 (82.45)	1,037 (82.37)		−0.002
Yes	1,385 (29.42)	225 (9.99)	1,160 (47.25)		0.746	443 (17.59)	221 (17.55)	222 (17.63)		0.002
COPD, n (%)				0.152					0.350	
No	4,443 (94.39)	2,137 (94.89)	2,306 (93.93)		−0.040	2,371 (94.16)	1,180 (93.73)	1,191 (94.60)		0.039
Yes	264 (5.61)	115 (5.11)	149 (6.07)		0.040	147 (5.84)	79 (6.27)	68 (5.40)		−0.039
Therapy										
CRRT, n (%)				0.733					0.585	
No	4,536 (96.37)	2,168 (96.27)	2,368 (96.46)		0.010	2,431 (96.54)	1,218 (96.74)	1,213 (96.35)		−0.021
Yes	171 (3.63)	84 (3.73)	87 (3.54)		−0.010	87 (3.46)	41 (3.26)	46 (3.65)		0.021
Vasoactive agent, n (%)				**<0.001**					0.935	
No	2,610 (55.45)	1,490 (66.16)	1,120 (45.62)		−0.412	1,522 (60.44)	762 (60.52)	760 (60.37)		−0.003
Yes	2,097 (44.55)	762 (33.84)	1,335 (54.38)		0.412	996 (39.56)	497 (39.48)	499 (39.63)		0.003
Ventilation, n (%)				**<0.001**					0.701	
No	3,001 (63.76)	1,624 (72.11)	1,377 (56.09)		−0.323	1,709 (67.87)	850 (67.51)	859 (68.23)		0.015
Yes	1,706 (36.24)	628 (27.89)	1,078 (43.91)		0.323	809 (32.13)	409 (32.49)	400 (31.77)		−0.015

Wbc, white blood cell; Bun, blood urea nitrogen; PTT, partial thromboplastin time; PT, prothrombin time; Po2, partial pressure of oxygen; Pco2, partial pressure of carbon dioxide; Spo2, saturation of peripheral oxygen; SBP, systolic blood pressure; DBP, diastolic blood pressure; Resp Rate Mean, respiratory rate mean; SOFA, Sepsis-related Organ Failure Assessment; GCS, Glasgow Coma Scale; APSIII, Acute Physiology Score III; OASIS, Oxford Acute Severity of Illness Score; CCI, charlson comorbidity index; COPD, chronic obstructive pulmonary disease; CRRT, continuous renal replacement therapy.

### Aspirin exposure and mortality outcomes

We constructed three cox proportional hazards models adjusted for potential confounding factors. Interestingly, the in-hospital, 30-day, 60-day, 90-day, and 180-day mortality rate in the aspirin group was consistently significant lower than that in the no aspirin group. Table 2 summarizes the detailed associations between the aspirin exposure and mortality rate. Specifically, compared to the no aspirin group, the in-hospital mortality decreased 41% before PSM (HR = 0.59, 95%CI [0.49 ~ 0.70], *p* < 0.001) and 44% after PSM (HR = 0.56, 95%CI [0.46 ~ 0.69], *p* < 0.001), the 30-day mortality decreased 38% before PSM (HR = 0.62, 95%CI [0.53 ~ 0.73], *p* < 0.001) and 36% after PSM (HR = 0.64, 95%CI [0.54 ~ 0.77], *p* < 0.001), the 60-day mortality decreased 34% before PSM (HR = 0.66, 95%CI [0.58 ~ 0.76], *p* < 0.001) and 33% after PSM (HR = 0.67, 95%CI [0.57 ~ 0.78], *p* < 0.001), the 90-day mortality decreased 31% before PSM (HR = 0.69, 95%CI [0.61 ~ 0.79], *p* < 0.001) and 31% after PSM (HR = 0.69, 95%CI [0.60 ~ 0.81], *p* < 0.001), and the 180-day mortality decreased 27% before PSM (HR = 0.73, 95%CI [0.64 ~ 0.82], *p* < 0.001) and 27% after PSM (HR = 0.73, 95%CI [0.63 ~ 0.83], *p* < 0.001) in the aspirin group. These results indicated that aspirin administration was an independent protective factor for in-hospital, 30-day, 60-day, 90-day, and 180-day mortality in SAE.

**Table 2:** Association between aspirin therapy and mortality of SAE patients.

Outcome	Before PSM						After PSM					
	Total (*n* = 4,707)	No aspirin (*n* = 2,252)	Aspirin (*n* = 2,455)	Model	HR (95% CI)	*p*	Total (*n* = 2,518)	No aspirin (*n* = 1,259)	Aspirin (*n* = 1,259)	Model	HR	*p*
In-hospital mortality, n (%)	683 (14.51)	387 (17.18)	296 (12.06)	1	0.70 (0.60 ~ 0.81)	<0.001	409 (16.24)	230 (18.27)	179 (14.22)	1	0.68 (0.56 ~ 0.82)	<0.001
				2	0.50 (0.42 ~ 0.59)	<0.001				2	0.65 (0.53 ~ 0.80)	<0.001
				3	0.59 (0.49 ~ 0.70)	<0.001				3	0.56 (0.46 ~ 0.69)	<0.001
30-day mortality, n (%)	872 (18.53)	507 (22.51)	365 (14.87)	1	0.63 (0.55 ~ 0.72)	<0.001	523 (20.77)	302 (23.99)	221 (17.55)	1	0.70 (0.59 ~ 0.83)	<0.001
				2	0.50 (0.43 ~ 0.59)	<0.001				2	0.69 (0.58 ~ 0.82)	<0.001
				3	0.62 (0.53 ~ 0.73)	<0.001				3	0.64 (0.54 ~ 0.77)	<0.001
60-day mortality, n (%)	1,078 (22.90)	604 (26.82)	474 (19.31)	1	0.68 (0.60 ~ 0.76)	<0.001	639 (25.38)	362 (28.75)	277 (22.00)	1	0.72 (0.62 ~ 0.85)	0.002
				2	0.54 (0.47 ~ 0.62)	<0.001				2	0.71 (0.61 ~ 0.83)	<0.001
				3	0.66 (0.58 ~ 0.76)	<0.001				3	0.67 (0.57 ~ 0.78)	<0.001
90-day mortality, n (%)	1,201 (25.52)	659 (29.26)	542 (22.08)	1	0.71 (0.63 ~ 0.79)	<0.001	713 (28.32)	397 (31.53)	316 (25.10)	1	0.75 (0.65 ~ 0.87)	0.001
				2	0.57 (0.50 ~ 0.65)	<0.001				2	0.74 (0.64 ~ 0.86)	<0.001
				3	0.69 (0.61 ~ 0.79)	<0.001				3	0.69 (0.60 ~ 0.81)	<0.001
180-day mortality, n (%)	1,405 (29.85)	757 (33.61)	648 (26.40)	1	0.73 (0.66 ~ 0.81)	<0.001	828 (32.88)	454 (36.06)	374 (29.71)	1	0.77 (0.67 ~ 0.89)	0.001
				2	0.60 (0.54 ~ 0.68)	<0.001				2	0.76 (0.66 ~ 0.87)	<0.001
				3	0.73 (0.64 ~ 0.82)	<0.001				3	0.73 (0.63 ~ 0.83)	<0.001

SAE, sepsis-associated encephalopathy; PSM, propensity score matching; HR, Hazard Ratio; CI, Confidence Interval.

Model 1: Unadjusted.

Model 2: Adjust for Age, Gender, Race, Weight.

Model 3: Adjust for Age, Gender, Race, Weight, wbc, hemoglobin, platelet, glucose, sodium, potassium, calcium, chloride, creatinine, aniongap, bun, PTT, PT, po2, pco2, base excess, spo2 mean, sbp mean, dbp mean, heart rate mean, resp rate mean, temperature mean, SOFA, GCS, APSIII, OASIS, CCI, Hypertension, Diabetes, Heart failure, Cardiovascular disease, COPD, CRRT, Vasoactive agent, Ventilation.

**Figure 3:**
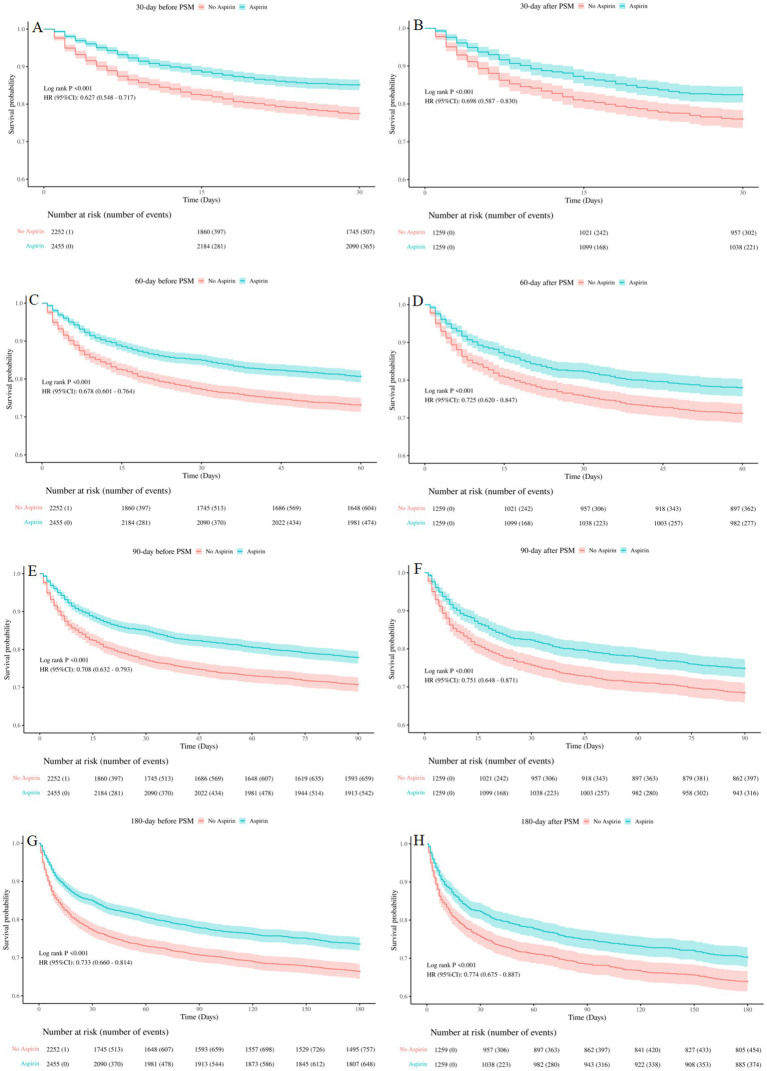
Kaplan-Meier survival curves between the aspirin group and no aspirin group **(A,B)**: 30-day survival curves before and after PSM, **(C,D)**: 60-day survival curves before and after PSM, **(E,F)**: 90-day survival curves before and after PSM, **(G,H)**: 180-day survival curves before and after PSM. no aspirin, no aspirin group; aspirin, aspirin group.

Kaplan–Meier curves showed that SAE patients who received aspirin therapy had significantly higher survival rate at 30-day, 60-day, 90-day, and 180-day, compared to those who did not receive aspirin (log-rank test: all *p* < 0.001) (Figure 3). These results emphasized the potential benefit of aspirin in improving survival outcomes for SAE patients.

### Aspirin exposure and composite outcomes

The result of Mann–Whitney U test showed that, the median length of hospital stay was significantly longer in the aspirin group compared to the no aspirin group before PSM (8.91 vs. 8.08, *p* < 0.001) and after PSM (9.10 vs. 8.02, *p* < 0.001). Additionally, we evaluated the risk of gastrointestinal hemorrhage between the two groups using the chi-square test, which revealed no significant differences both before PSM (*p* = 0.10) and after PSM (*p* = 0.37) (Table 3).

**Table 3:** Composite outcomes of the aspirin group and no aspirin group.

Outcome	Before PSM				After PSM			
	Total (*n* = 4,707)	No aspirin (*n* = 2,252)	Aspirin (*n* = 2,455)	*p*	Total (*n* = 2,518)	No aspirin (*n* = 1,259)	Aspirin (*n* = 1,259)	*p*
Length of hospital, M (Q₁, Q₃)	8.61 (5.54, 14.21)	8.08 (4.92, 14.58)	8.91 (5.90, 13.96)	<0.001	8.67 (5.31, 14.74)	8.02 (4.91, 13.89)	9.10 (5.75, 15.69)	<0.001
Gastrointestinal hemorrhage, n (%)	23 (0.49)	15 (0.67)	8 (0.33)	0.10	11 (0.44)	7 (0.56)	4 (0.32)	0.37

### Subgroup analysis

**Figure 4:**
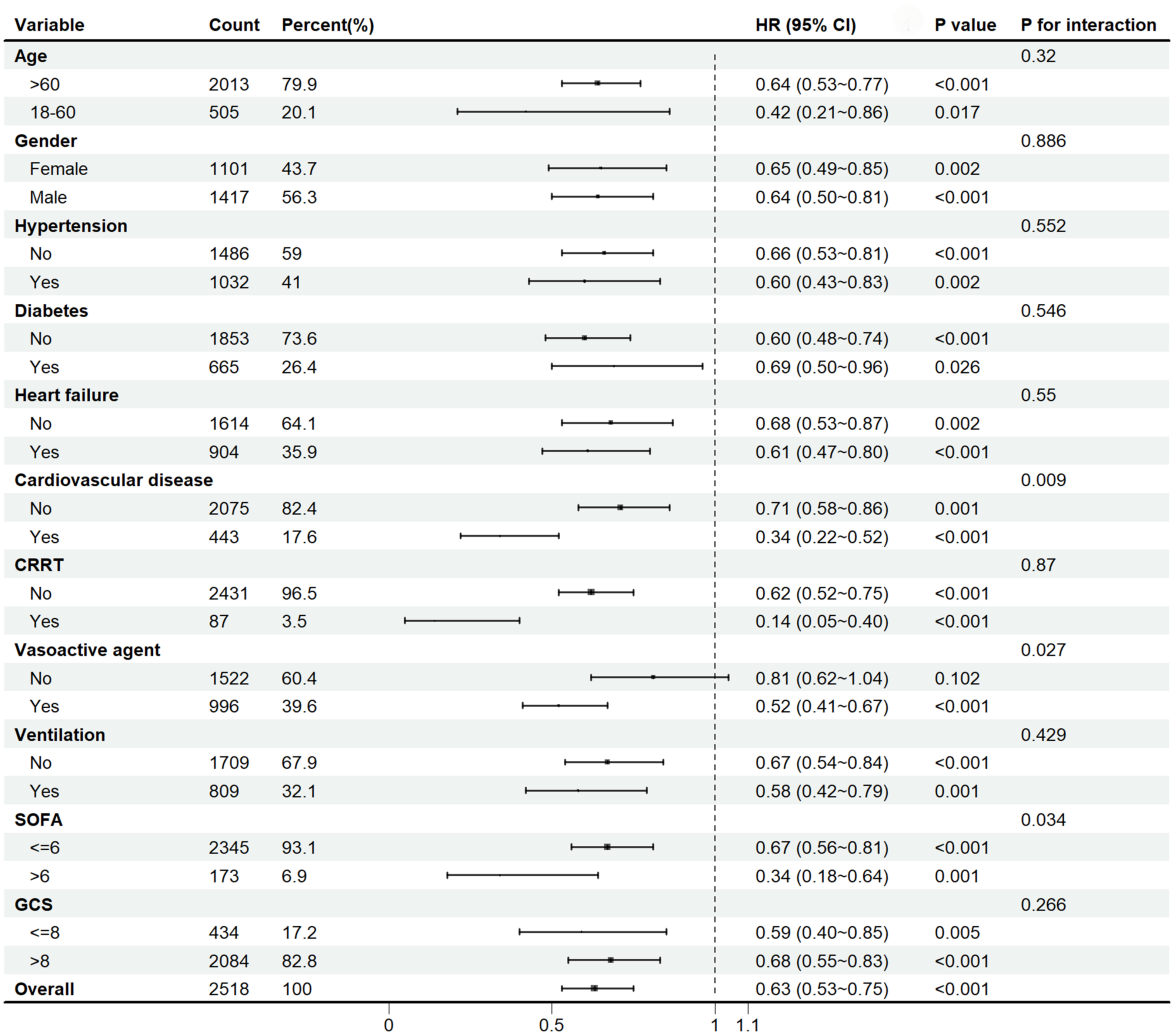
Subgroup analysis of the 30-day mortality in SAE patients. HR, hazard ratio; CI, confidence interval; CRRT, continuous renal replacement therapy; SOFA, Sepsis-related Organ Failure Assessment; GCS, Glasgow Coma Scale.

The subgroup analysis was applied based on age, gender, hypertension, diabetes, heart failure, cardiovascular disease, CRRT, vasoactive agent, ventilation, SOFA score, and GCS score. The results indicated that aspirin therapy was significantly associated with a reduction in 30-day mortality in the overall population (HR 0.63, 95%CI [0.53–0.75], *p* < 0.001), as depicted in the Forest Plot in Figure 4. It seemed that aspirin’s protective effect was more pronounced in the following subgroups: age < 60 (HR = 0.42, 95%CI [0.21 ~ 0.86], *p* = 0.017), male (HR = 0.64, 95%CI [0.50 ~ 0.81], *p* < 0.001), hypertension (HR = 0.60, 95%CI [0.43 ~ 0.83], *p* = 0.002), non-diabetes (HR = 0.60, 95%CI [0.48 ~ 0.74], *p* < 0.001), heart failure (HR = 0.61, 95%CI [0.47 ~ 0.80], *p* < 0.001), cardiovascular disease (HR = 0.34, 95%CI [0.22 ~ 0.52], *p* < 0.001), CRRT (HR = 0.14, 95%CI [0.05 ~ 0.40], *p* < 0.001), ventilation (HR = 0.58, 95%CI [0.42 ~ 0.79], *p* = 0.001), SOFA>6 (HR = 0.34, 95%CI [0.18 ~ 0.64], *p* = 0.001), and GCS ≤ 8 (HR = 0.59, 95%CI [0.40 ~ 0.85], *p* = 0.005). Aspirin reduced 30-day mortality in the subgroup receiving vasoactive agent treatment by 48% (HR = 0.52, 95%CI [0.41 ~ 0.67], *p* < 0.001), whereas it did not reduced the mortality in those without receiving vasoactive agent treatment. An interaction effect was observed in the SOFA score. Aspirin intervention reduced 30-day mortality by 33% (HR = 0.67, 95%CI [0.56 ~ 0.81], *p* < 0.001) in patients with SOFA<=6 versus 66% (HR = 0.34, 95%CI [0.18 ~ 0.64], *p* = 0.001) in those with SOFA>6 (p for interaction =0.034). Our subgroup analysis might contribute to the personalization of aspirin treatment for SAE patients.

### Sensitivity analysis

We calculated the *E*-value to assess the association between the mortality and aspirin administration. The result indicated that association between 30-day mortality and aspirin would require an unmeasured confounder with a relative risk greater than 2.50 to account for the observed effect. Therefore, we considered that the residual confounders could explain the observed association, and it appeared that other unknown or unmeasured confounders exerted a relatively smaller effect on 30-day mortality compared to known risk factors. Similarly, the other unknown or unmeasured factors also had a relatively minor impact on in-hospital (*E*-value>2.97), 60-day (*E*-value>2.35), 90-day (*E*-value>2.26), and 180-day mortality (*E*-value>2.08) (Table 4).

**Table 4:** *E*-value for mortality association in aspirin-treated SAE patients.

Outcomes	*E*-value	Upper limit of 95% CI
In-hospital mortality	2.97	2.26
30-day mortality	2.50	1.92
60-day mortality	2.35	1.88
90-day mortality	2.26	1.77
180-day mortality	2.08	1.70

### Different aspirin exposure timing and outcome

**Figure 5:**
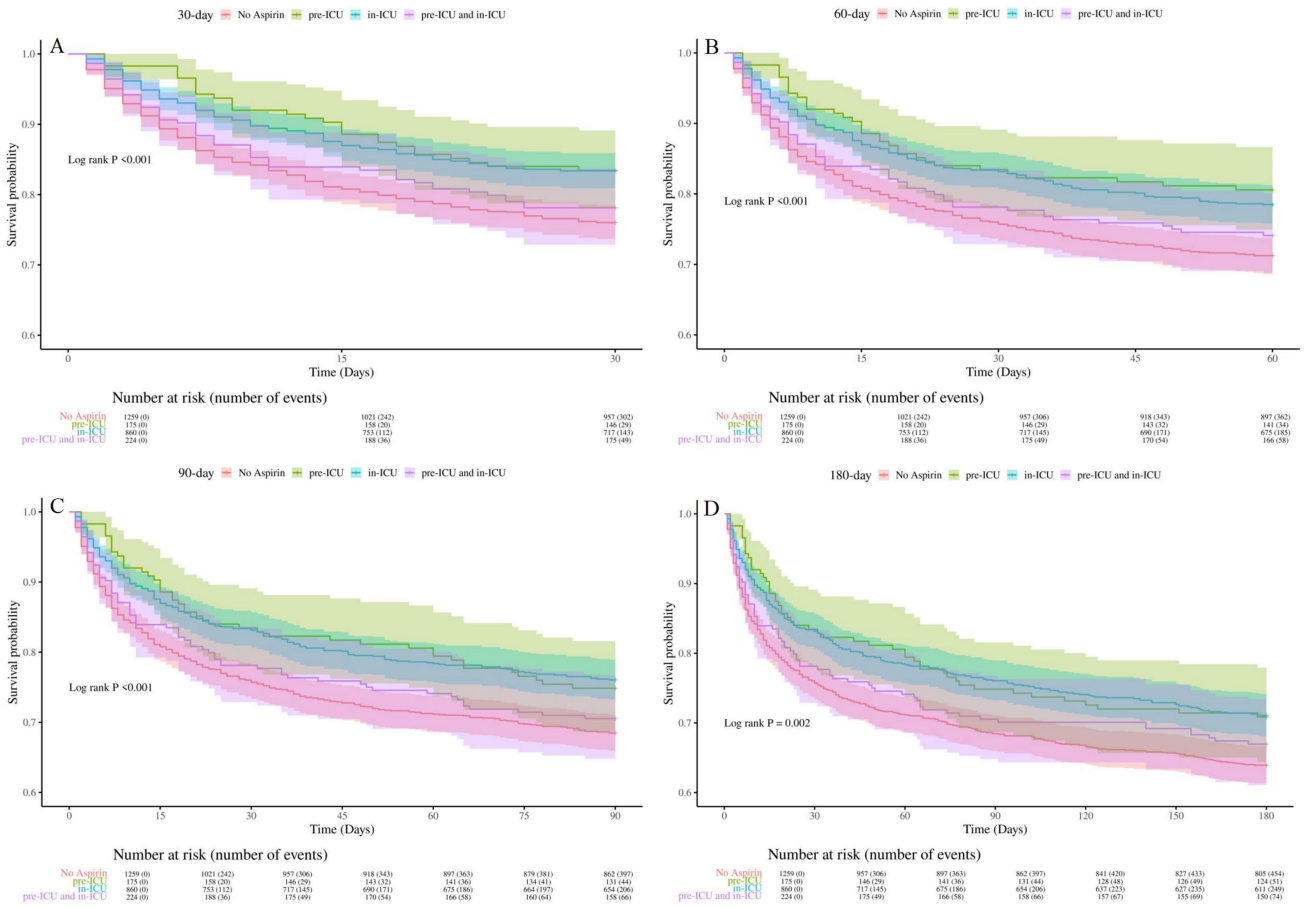
Kaplan-Meier survival curves for groups of different aspirin exposure timing **(A)**: 30-day survival curves for groups of different aspirin exposure timing, **(B)**: 60-day survival curves for groups of different aspirin exposure timing, **(C)** 90-day survival curves for groups of different aspirin exposure timing, **(D)**: 180-day survival curves for groups of different aspirin exposure timing. No Aspirin, no aspirin group; pre-ICU, aspirin pre-ICU group; in-ICU, aspirin in-ICU group; pre-ICU and in-ICU, aspirin pre-ICU and in-ICU group.

To determine the optimal treatment timing, we divided SAE patients into four groups: aspirin pre-ICU group, aspirin in-ICU group, aspirin pre-ICU and in-ICU group, and no aspirin group. The multifactorial cox proportional hazards models demonstrated that, compared to the no aspirin group, the in-hospital, 30-day, 60-day, 90-day, and 180-day mortality rate of SAE patients in the other three groups decreased significantly (Table 5). As illustrated in Figure 5, Kaplan–Meier curves suggested the survival of SAE patients in pre-ICU group, in-ICU group, and pre-ICU and in-ICU group was higher than that in no aspirin group for 30-day, 60-day, 90-day, and 180-day (log-rank test: all *p* < 0.05).

**Table 5:** Association between aspirin exposure timing and mortality of SAE patients.

Outcome	Total (*n* = 2,518)	No aspirin (*n* = 1,259)	pre-ICU (*n* = 175)	HR (95% CI)	*p*	in-ICU (*n* = 860)	HR (95% CI)	*p*	pre-ICU and in-ICU (*n* = 224)	HR (95% CI)	*p*
In-hospital mortality, n (%)	409 (16.24)	230 (18.27)	117 (13.60)	0.53 (0.34 ~ 0.82)	0.005	26 (14.86)	0.57 (0.45 ~ 0.72)	<0.001	36 (16.07)	0.57 (0.40 ~ 0.83)	0.003
30-day mortality, n (%)	523 (20.77)	302 (23.99)	143 (16.63)	0.56 (0.38 ~ 0.85)	0.006	29 (16.57)	0.64 (0.52 ~ 0.79)	<0.001	49 (21.88)	0.70 (0.52 ~ 0.96)	0.027
60-day mortality, n (%)	639 (25.38)	362 (28.75)	185 (21.51)	0.55 (0.38 ~ 0.80)	0.002	34 (19.43)	0.68 (0.57 ~ 0.82)	<0.001	58 (25.89)	0.70 (0.53 ~ 0.93)	0.015
90-day mortality, n (%)	713 (28.32)	397 (31.53)	206 (23.95)	0.67 (0.48 ~ 0.92)	0.015	44 (25.14)	0.69 (0.58 ~ 0.82)	<0.001	66 (29.46)	0.72 (0.55 ~ 0.94)	0.015
180-day mortality, n (%)	828 (32.88)	454 (36.06)	249 (28.95)	0.69 (0.51 ~ 0.93)	0.014	51 (29.14)	0.74 (0.63 ~ 0.87)	<0.001	74 (33.04)	0.70 (0.55 ~ 0.91)	0.006

The multifactorial cox proportional hazards model was adjust for Age, Gender, Race, Weight, wbc, hemoglobin, platelet, glucose, sodium, potassium, calcium, chloride, creatinine, aniongap, bun, PTT, PT, po2, pco2, base excess, spo2 mean, sbp mean, dbp mean, heart rate mean, resp rate mean, temperature mean, SOFA, GCS, APSIII, OASIS, CCI, Hypertension, Diabetes, Heart failure, Cardiovascular disease, COPD, CRRT, Vasoactive agent, Ventilation.

### Different aspirin dosage and outcome

To further investigate the effects of different aspirin dosages on mortality, we divided the SAE patients into two groups: low-dose aspirin group (≤300 mg/day) and high-dose aspirin group (>300 mg/day). Then, three cox proportional hazards model was used to evaluate mortality rate. We discovered that patients in the high-dose aspirin group experienced a significantly higher mortality rate compared to those in the low-dose group for in-hospital, 30-day, 60-day, 90-day and 180-day, as detailed in Table 6.

**Table 6:** Association between aspirin dosage and mortality of SAE patients.

Outcome	Total (*n* = 1,259)	Low dose aspirin (*n* = 907)	High dose aspirin (*n* = 352)	Model	HR (95% CI)	*p*
In-hospital mortality, n (%)	179 (14.22)	110 (12.13)	69 (19.60)	1	1.54 (1.14 ~ 2.09)	0.005
				2	1.61 (1.17 ~ 2.20)	0.003
				3	1.48 (1.06 ~ 2.07)	0.023
30-day mortality, n (%)	221 (17.55)	141 (15.55)	80 (22.73)	1	1.52 (1.15 ~ 1.99)	0.003
				2	1.55 (1.17 ~ 2.06)	0.002
				3	1.35 (1.01 ~ 1.82)	0.049
60-day mortality, n (%)	277 (22.00)	178 (19.63)	99 (28.12)	1	1.51 (1.18 ~ 1.93)	0.001
				2	1.54 (1.20 ~ 1.98)	<0.001
				3	1.33 (1.02 ~ 1.74)	0.033
90-day mortality, n (%)	316 (25.10)	205 (22.60)	111 (31.53)	1	1.48 (1.17 ~ 1.86)	<0.001
				2	1.54 (1.21 ~ 1.95)	<0.001
				3	1.32 (1.03 ~ 1.69)	0.030
180-day mortality, n (%)	374 (29.71)	245 (27.01)	129 (36.65)	1	1.45 (1.17 ~ 1.80)	<0.001
				2	1.49 (1.20 ~ 1.86)	<0.001
				3	1.26 (1.01 ~ 1.59)	0.045

SAE, sepsis-associated encephalopathy; HR, Hazard Ratio; CI, Confidence Interval.

Model 1: Unadjusted.

Model 2: Adjust for Age, Gender, Race, Weight.

Model 3: Adjust for Age, Gender, Race, Weight, wbc, hemoglobin, platelet, glucose, sodium, potassium, calcium, chloride, creatinine, aniongap, bun, PTT, PT, po2, pco2, base excess, spo2 mean, sbp mean, dbp mean, heart rate mean, resp rate mean, temperature mean, SOFA, GCS, APSIII, OASIS, CCI, Hypertension, Diabetes, Heart failure, Cardiovascular disease, COPD, CRRT, Vasoactive agent, Ventilation.

**Figure 6:**
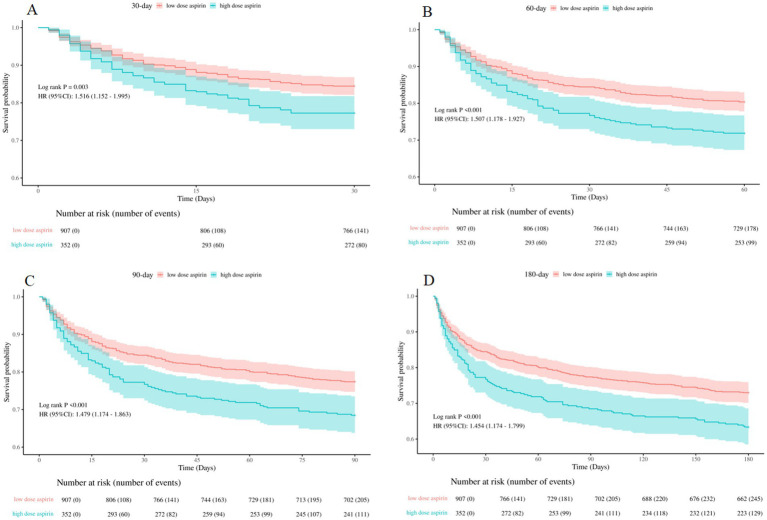
Kaplan–Meier survival curves between the low-dose and high-dose aspirin group **(A)**: 30-day survival curves between the low-dose and high-dose aspirin group, **(B)**: 60-day survival curves between the low-dose and high-dose aspirin group, **(C)**: 90-day survival curves between the low-dose and high-dose aspirin group, **(D)**: 180-day survival curves between the low-dose and high-dose aspirin group. low dose aspirin, low-dose aspirin group; high dose aspirin, high-dose aspirin group.

The Kaplan–Meier curves displayed in Figure 6 demonstrated that patients in the high-dose group exhibited a significantly higher mortality rate for 30-day, 60-day, 90-day, and 180-day, compared to those in the low-dose group (log-rank test: all *p* < 0.05).

## Discussion

Sepsis was a complex syndrome caused by a exaggerated inflammatory response to pathogens (26). In this condition, excessive platelet activation triggered thrombosis in the microvasculature, ultimately leading to multiple organ failure and potentially death (27). Sepsis-Associated Encephalopathy (SAE) was one of the most severe complications of sepsis, and clinical management for SAE was mainly focused on symptomatic and supportive care. The pathogenesis of SAE remained poorly understood, and the neuroinflammation was one of the critical pathogenic mechanisms of SAE (28–30).

Aspirin has become as a potential therapeutic option of sepsis and its complication. It could alleviated severe systemic inflammatory responses through multiple mechanisms, including antiplatelet effects, anti-inflammatory properties, COX inhibition, nitric oxide release, and modulation of the nuclear factor kappa B (NF-κB) pathway (31, 32). Researches showed that aspirin therapy significantly decreased vegetation weight, echocardiographic vegetation growth, and bacterial densities in both vegetation and kidneys in a rabbit model of *S. aureus* endocarditis (33). Aspirin therapy was associated with a lower 90-day mortality rate in septic patients, as indicated by data from the National Health Insurance Research Database of Taiwan (32). Additionally, aspirin has been found to influence various pathogenic mechanisms involved in sepsis and ARDS, as demonstrated by vitro studies, animal research, and observational analyses (12). According to Yu et al., aspirin helped reduce ARDS by inhibiting pulmonary inflammation through the NF-κB pathway (34). Chen et al. discovered that aspirin could shorten the ICU stay and reduce both 30-day and 90-day mortality in patients with sepsis-associated acute kidney injury (14). Moreover, Yiming et al. found that aspirin use was linked to lower 28-day, 90-day and 1-year mortality in patients with sepsis-induced myocardial injury, regardless of the timing of aspirin administration (19).

Our study investigated the efficacy of aspirin on the prognosis of SAE. In this study, 2,455 (52.2%) patients before PSM and 1,259 (50%) patients after PSM received aspirin were enrolled. The adjusted models demonstrated that aspirin treatment significantly reduced the 30-day mortality risk, as well as in-hospital, 60-day, 90-day, and 180-day mortality risk among SAE patients. The results indicated that aspirin was an independent protective factor for SAE. Aspirin may exert beneficial effects through their pleiotropic properties, especially anti-inflammatory effects (13). Our findings was consistent with previous research. However, patients in the aspirin group experienced a longer hospital stay compared to those in the no aspirin group. This extended length of stay could be explained by the higher mortality rate observed in the no aspirin group. Specifically, patients in the no aspirin group who died earlier during their hospitalization contributed to a shorter overall average hospital stay for this group. In contrast, patients in the aspirin group with lower mortality rates remained hospitalized for a longer period, which ultimately led to longer hospital stay for this group. Our analysis showed no significant difference in the incidence of gastrointestinal hemorrhage between the two groups. Given the small number of patients with gastrointestinal hemorrhage, the potential for statistical bias affecting the negative result could not be excluded, and further large-scale studies were needed to validate this finding. All the results proved the potential benefits and safety of aspirin in improving clinical outcomes for SAE patients.

Aspirin was usually used as a secondary prevention and treatment strategy for cardiovascular and cerebrovascular diseases. In our subgroup analysis, aspirin appeared to be more effective in SAE patients with hypertension, heart failure, and cardiovascular disease, possibly related to its antiplatelet aggregation properties. Furthermore, aspirin showed better effectiveness in SAE patients with CRRT, vasoactive agent, ventilation, SOFA>6, and GCS < 8. This might be attributed to the fact that, patients experiencing more serious illness had a more severe inflammatory response, and aspirin could better exert its anti-inflammatory effects. These findings suggest that aspirin may play a crucial role on SAE, especially for the patients with complex comorbidities and severe conditions, emphasizing the importance of personalized treatment strategies in this vulnerable population.

The efficacy among different aspirin exposure timing groups was evaluated. The results suggested that the mortality rate for the in-hospital, 30-day, 60-day, 90-day, and 180-day decreased in the three aspirin administration group. The beneficial effect persisted regardless of whether aspirin was used before ICU, in ICU, or both before and in ICU. The significant reduction in both short-term and long-time mortality had great importance for guiding clinical strategies.

The efficacy of different aspirin dosage in SAE patients was also assessed. The results indicated that the high-dose group had increased mortality rate for the in-hospital, 30-day, 60-day, 90-day, and 180-day compared to those receiving low-dose aspirin. Our discovery was consistent with previous research (9, 15). Low-dose aspirin functioned by inhibiting cyclooxygenase-1 (COX-1), while high-dose aspirin influenced the COX-2 and NF-κB pathways (31, 35, 36). Our study showed that SAE patients receiving low-dose aspirin experienced greater benefits. One possible explanation was that patients in the high-dose aspirin group might had more severe conditions at baseline. Additionally, differences in individual responsiveness to aspirin between the two groups could be another contributing factor, rather than a direct effect of aspirin dosage on specific physiological pathways.

There were several limitations on this study. First, we demonstrated an association between aspirin and prognosis of SAE patients, but unable to elucidate the specific mechanisms of aspirin. Second, as an retrospective study, residual confounding factors could not be completely excluded, although PSM were used to mitigate this issue. Third, the MIMIC-IV database covered critically ill patients with a long time span from 2008 to 2022, and the diagnostic criteria might differ for each patient. Therefore, large-scale prospective studies were necessary to further investigate the relationship between aspirin and prognosis of SAE patients.

## Conclusion

Aspirin could reduce the mortality risk of SAE patients for in-hospital, 30-day, 60-day, 90-day, and 180-day, without increasing the risk of gastrointestinal hemorrhage. The benefits observed persisted regardless of aspirin exposure timing. Patients received high-dose aspirin exhibited a higher mortality risk compared to those in the low-dose group. Further researches are necessary to explore the specific mechanisms of aspirin on SAE patients.

## Data Availability

The original contributions presented in the study are included in the article/supplementary material, further inquiries can be directed to the corresponding authors.
